# Self-Directed Learning Versus Traditional Teaching in Learning Gross Anatomy Among First-Year Medical Students: A Comparative Study

**DOI:** 10.7759/cureus.66542

**Published:** 2024-08-09

**Authors:** Sharmistha Biswas, Nilima R Thosar, Phalguni Srimani

**Affiliations:** 1 Department of Anatomy, Calcutta National Medical College, Kolkata, IND; 2 Department of Pediatric and Preventive Dentistry, Sharad Pawar Dental College and Hospital, Datta Meghe Institute of Higher Education and Research (Deemed to be University), Wardha, IND

**Keywords:** liver, gross anatomy, medical education, traditional teaching methods, self-directed learning (sdl)

## Abstract

Introduction: Gross anatomy is the first subject to be grasped by budding medicos before learning a long series of skills and competencies required to bloom as a physician or surgeon. In recent years, the teaching time for anatomy has been reduced. The number of anatomy teachers is much less in comparison to the increasing number of students. There is also a paradigm shift in medical education; it has become competency-based, learner-centric, and technology-based. So, anatomical education is also shifting to adopt blended learning strategies. Now, the onus of learning lies more with the students, which can be promoted through self-directed learning (SDL). Many first-year medical students are unprepared for SDL and need assistance understanding SDL. Structured SDL can successfully instill the habit of SDL in young medical students, who are supposed to be lifelong learners in their professional careers. The concept of structured SDL under the supervision of teachers is comparatively new in India. Very few studies are there to compare the effectiveness of SDL with those of traditional teaching methods.

The present study aimed to evaluate the effectiveness of SDL in learning the gross anatomy of an important viscus, viz., the liver, and to compare it with the effectiveness of the traditional method of teaching by demonstration/prosection.

Methods: This interventional comparative study was carried out at the department of anatomy of a government medical college in Kolkata, India. Sixty willing first-year undergraduate medical students were included in the study. The students were divided into two batches (Batch A and Batch B) of 30 each. Students of Batch A were taught the viscus (liver) by the traditional method of teaching by demonstration/prosection for one and a half hours. Validated pre-test and post-test questionnaires were administered to evaluate learning outcomes. Students of Batch B had a structured self-directed learning (SDL) session under the supervision of teachers on the same topic, viz., viscus (liver), for one and a half hours. The pre-test and post-test questionnaires were given before and after the SDL sessions to evaluate learning outcomes. The results were statistically analyzed.

Results: It was observed that the mean post-test score after traditional teaching improved significantly compared to the mean pre-test score. Also, the mean post-test score after the SDL session improved significantly compared to the mean pre-test score. It was found that the mean post-test score after exposure to traditional teaching methods was slightly better than the mean post-test scores after exposure to SDL, but this difference was not statistically significant.

Conclusion: It can be inferred that structured SDL under the supervision of teachers was almost as good as the traditional teaching method by demonstration/prosection in learning the gross anatomy of an important viscus, viz., liver. In the context of the reduced number of anatomy teachers and an increasing number of students, this may open up an option of teaching some portion of gross anatomy, like a few viscera, by SDL. However, a more robust study with a larger sample size can be more conclusive.

## Introduction

Gross anatomy teaches the morphology of the human body. It is the first subject to be grasped by budding medicos before learning a long series of skills and competencies required to bloom as a physician or surgeon [1]. The time and resources allocated to anatomy teaching have been reduced [2]. The number of anatomy teachers is much less when compared to the increased number of students. The teaching time for anatomy has decreased. There has also been a paradigm shift in medical education, becoming competency-based, learner-centric, and technology-based. So, anatomical education is also shifting to adopt blended learning strategies. Teachers have to deliver new curriculums to the vast and diverse student population through a more learner-centered approach [3]. Keeping pace with fast-progressing medical education, anatomical education also focused more on adopting blended learning strategies [4]. Medical students should acquire competencies through active learning, which can be promoted through self-directed learning (SDL). The responsibility of learning lies largely with the students. Structured self-directed learning can effectively instill the practice of self-directed learning in young medical students, who are anticipated to be lifelong learners in their career path [5]. Adult education authority Malcolm Knowles [6] describes self-directed learning (SDL) as an approach in which students independently identify their learning requirements, set their learning objectives, locate learning materials, and assess their learning achievements. Many first-year medical students may not be prepared for SDL. In the initial phases, students should get help, support, and direction to improve their participation [7]. To successfully inculcate the habit of SDL, younger learners should be assisted by helping them understand SDL.

The modification of the undergraduate medical curriculum occurred without comprehensive research. Medical schools worldwide vary significantly in how they present their curriculum. The anatomy curriculum has become integrated and more clinically relevant [8]. Implementing competency-based medical education (CBME) in India poses problems for professors and students due to the significant restructuring of traditional teaching methods [9]. As an alternate form of learning for knowledge acquisition, self-directed learning can be considered [10]. The competency or learning objectives should be carefully selected from the core or non-core topic of the curriculum considering that they are interesting for the students. Although identification of the topic is the learner's responsibility, first-year undergraduate students may not be capable of doing so. SDL sessions should be done in small groups of students. The facilitator can guide the group to refine their learning objectives and identify resources for the topic [11]. Learners need to pass through stages of increasing self-direction, and good teaching/facilitating matches the learner's stage of self-direction; thus, it can help them advance toward greater self-direction [12]. The concept of structured SDL under the supervision of teachers is comparatively new in India. A very limited number of studies are available to compare the effectiveness of SDL with that of traditional teaching methods. So, the current study aimed to evaluate the effectiveness of SDL in learning the gross anatomy of an important viscus, viz., the liver, and to compare it with the efficacy of the traditional teaching method by demonstration/prosection. The objectives are to evaluate learning outcomes after exposure to SDL, as well as the traditional demonstration/prosection method by pre-test and post-test scores, and to compare the effectiveness of self-directed learning (SDL) with the conventional method of teaching by demonstration/prosection in learning a viscus (liver) among first-year medical students by comparing post-test scores after exposure to both methods.

## Materials and methods

This interventional comparative study was conducted at the department of anatomy of a government medical college in Kolkata, India, from January 2024 to March 2024. The study was done after receiving approval from the Ethics Committee of Calcutta National Medical College (EC-CNMC/2024/388, dated 9/03/2024).

**Figure 1:**
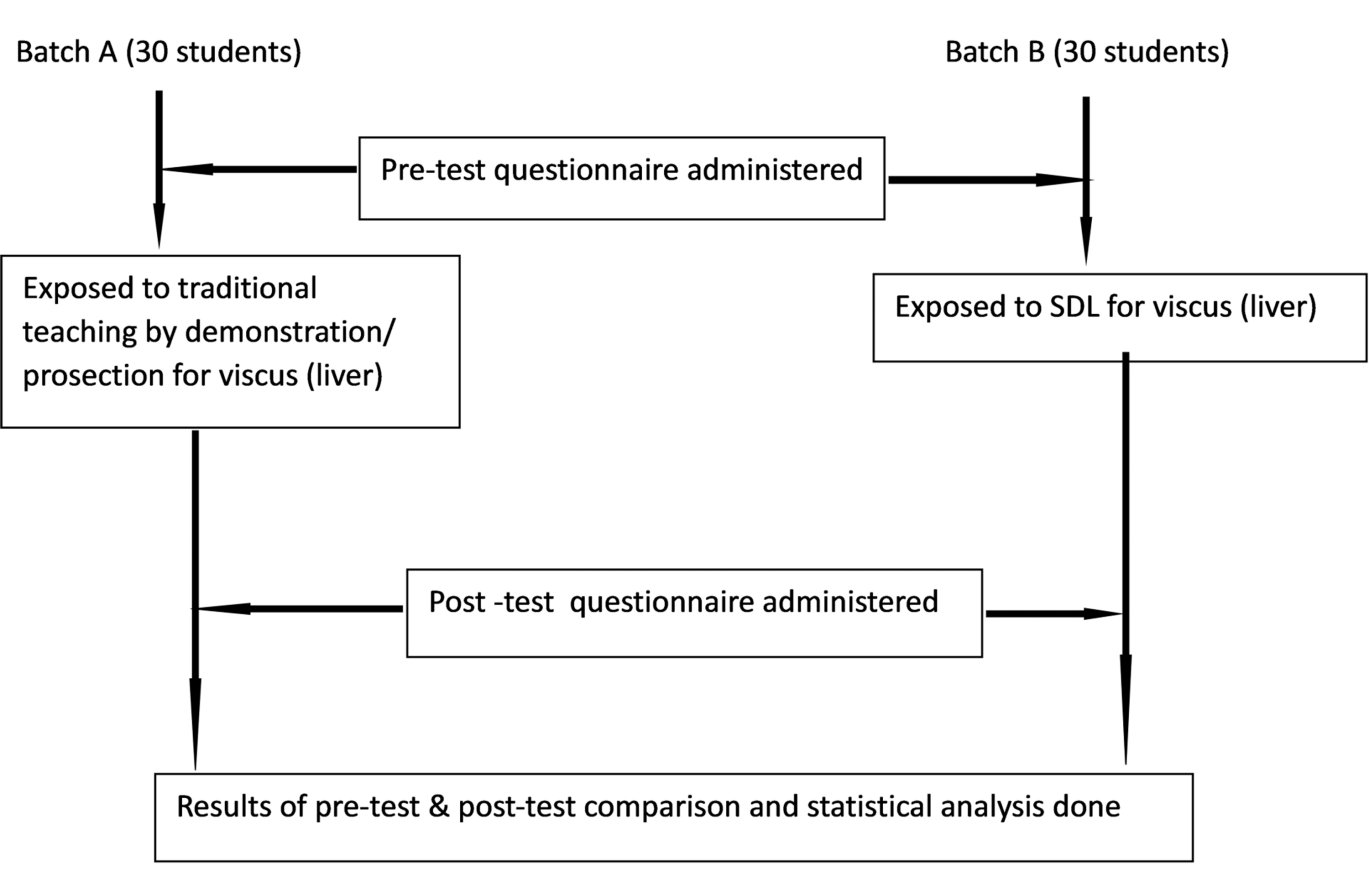
Flowchart for methods SDL: self-directed learning

In the medical college, the intake in first-year MBBS is 250 students. They are divided into four equal groups. All groups underwent the same study modules in the department of anatomy on different days. One group consisting of 60 first-year undergraduate medical students, who were willing and gave informed consent to participate in our study, was included in the study. The students who were unwilling or absent were excluded. The students were divided into two batches (Batch A and Batch B) of 30 students in each group. Students of Batch A were taught one important abdominal viscus, the liver, using the traditional teaching method by demonstration/prosection for two hours. A pre-test and post-test questionnaire, consisting of multiple-choice questions (MCQs) and short-answer questions (SAQs), carrying 10 marks, one mark each, was administered to evaluate the learning outcome of traditional teaching methods. The questionnaire was validated by two subject experts in anatomy and two members of the medical education unit of the medical college. Students of Batch B had a self-directed learning session on the same topic, viz., viscus (liver), for two hours. Students were instructed to bring their textbooks, reference materials, and laptops with internet connections to encourage them to do computer-assisted learning. YouTube video links were provided with LCD screens so they could learn from the video. Pre-test and post-test questionnaires were given just before and after the SDL sessions to evaluate the learning outcomes of the SDL method (Figure 1). The two sessions were held at the same time in two classrooms. Before the sessions were held, the gross anatomy of the liver was not taught by didactic lectures. All students of the other three groups had similar sessions of traditional teaching and SDL but were not included in the study. Thus, selection bias was overcome.

The scores of pre-test and post-test MCQ questionnaires of the sessions were compared for both methods. The results of the post-test MCQ questionnaire for both methods were compared.

Data were collected manually. Data analysis was done statistically with the help of Prism software. Paired t-test was done to compare the means of the scores. P < 0.05 was considered statistically significant.

## Results

The scores of the pre-test taken before starting the session of teaching the viscus (liver) by traditional methods of demonstration/prosection, as well as the scores of the post-test after the session, were recorded (Table 1).

**Table 1:** Pre-test and post-test scores for traditional teaching of the liver

Pre-test scores	Post-test scores
6	9
5	9
5	9
6	7
9	10
5	10
9	10
9	10
9	9
4	10
8	10
4	8
8	10
5	9
9	10
8	10
9	10
5	10
7	10
6	9
6	9
8	9
9	9
9	10
5	9
1	5
4	7
3	8
4	9
4	5

They were compared and analyzed statistically using a paired t-test. It was observed that the mean pre-test score before traditional teaching was 6.30 (standard deviation (SD): 2.25), whereas the mean post-test score was 8.97 (SD: 1.38). The P value is less than 0.0001 (Table 2).

**Table 2:** Pre-test and post-test data for traditional teaching P < 0.05 is considered significant. SD: standard deviation, SEM: standard error of the mean

Group	Traditional teaching (pre-test)	Traditional teaching (post-test)
Mean of scores	6.30	8.97
SD	2.25	1.38
SEM	0.41	0.25
Number of participants	30	30
P < 0.0001		

The scores of the pre-test taken before starting the SDL session for the liver, as well as the scores of the post-test after the session, were recorded (Table 3).

**Table 3:** Pre-test and post-test scores for SDL of the liver SDL: self-directed learning

Pre-test scores	Post-test scores
6	10
5	10
9	10
5	10
7	10
5	8
4	8
8	7
9	9
6	7
6	8
7	9
9	10
9	8
6	9
8	8
5	8
6	6
8	9
7	8
7	10
6	10
8	8
7	8
5	8
5	6
2	8
5	9
3	9
3	8

The mean pre-test score before SDL was recorded to be 6.20 (SD: 1.86), whereas the mean post-test score was 8.53 (SD: 1.17). The P value is less than 0.0001 (Table 4).

**Table 4:** Pre-test and post-test data for SDL P < 0.05 is considered significant. SDL: self-directed learning, SD: standard deviation, SEM: standard error of the mean

Group	SDL (pre-test)	SDL (post-test)
Mean of scores	6.20	8.53
SD	1.86	1.17
SEM	0.34	0.21
Number of participants	30	30
P < 0.0001		

It was found that the mean post-test score after exposure to traditional teaching methods was 8.92 (SD: 1.38), and the mean post-test score after exposure to SDL was 8.53 (SD: 1.17). The P value is 0.1771 (Table 5).

**Table 5:** Post-test data for traditional teaching and SDL P < 0.05 is considered significant. SDL: self-directed learning, SD: standard deviation, SEM: standard error of the mean

Group	Traditional teaching (post-test)	SDL (post-test)
Mean of scores	8.97	8.53
SD	1.38	1.17
SEM	0.25	0.21
Number of participants	30	30
P = 0.1771		

## Discussion

**Figure 2:**
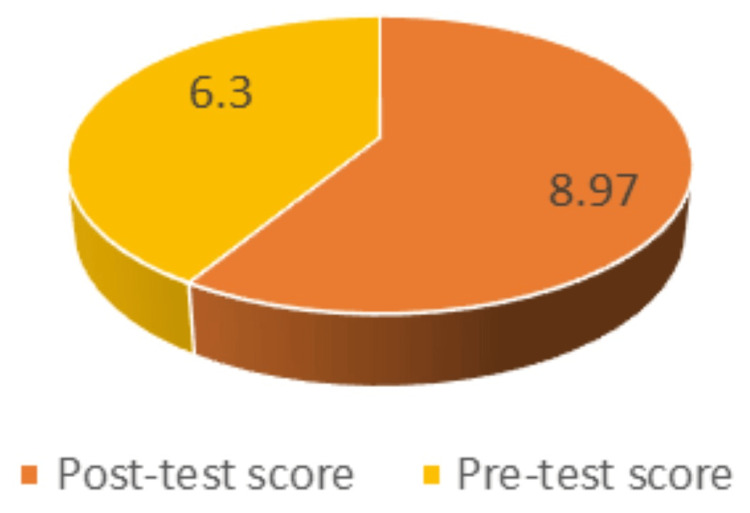
Mean pre-test score versus mean post-test score for traditional teaching

In the present study, using a paired t-test for the mean pre-test score and the mean post-test score before and after exposure to traditional teaching, the P value is <0.0001. By traditional standards, this is considered highly statistically significant (Figure 2).

It can, therefore, be remarked that students' learning outcome improves significantly after they are exposed to conventional teaching of gross anatomy of viscera through demonstration/prosection methods. The finding is congruent with that of the study by Collins [13], who noted that using prosected cadaver specimens maximizes learning. The dissected cadaver remains the most powerful means of learning; it has survived the test of time [14].

**Figure 3:**
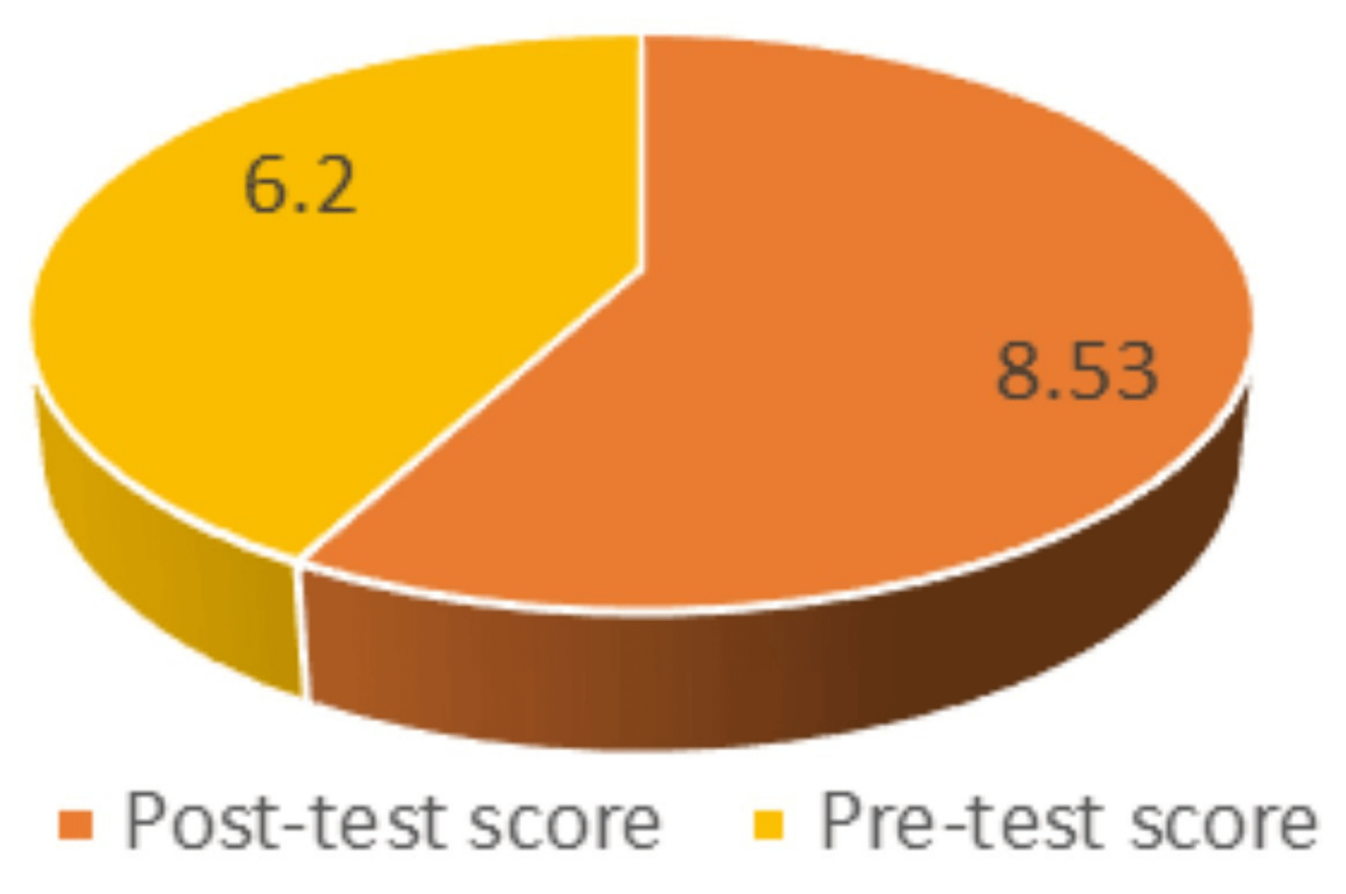
Mean pre-test score versus mean post-test score for SDL SDL: self-directed learning

For the mean pre-test score of students before exposure to SDL when compared by the paired t-test with the mean post-test score after exposure to SDL, the P value was found to be <0.0001. By conventional criteria, this is considered to be extremely statistically significant (Figure 3).

The observation suggests that SDL of the viscera's gross anatomy significantly improves learning outcomes. In a study conducted in the year 2018, students' assessment using pre-test and post-test multiple-choice questions (MCQs) after the SDL session showed that all students scored better in the post-test. Thus, it was evident that the SDL session had been very helpful in gathering knowledge. Research in medical education reports improvement in all learning domains after exposure to SDL [15,16].

**Figure 4:**
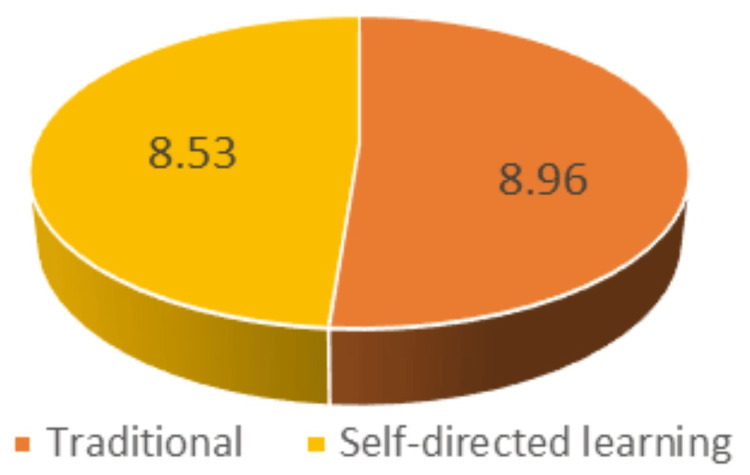
Mean post-test score for traditional teaching versus mean post-test score for SDL SDL: self-directed learning

It was found in our study that both methods of teaching the gross anatomy of viscera effectively improved learning outcomes. Although the mean post-test score after the traditional teaching method by dissection/prosection was greater than the mean post-test score after the SDL session, the difference between the two teaching-learning methods was insignificant (P > 0.05) (Figure 4).

Wilson et al. (2018) [17] had a similar view; they found that student performance scores were statistically equivalent when comparing traditional teaching/dissection to other laboratory methods.

For the success of SDL, the teacher's role must evolve as a facilitator, who should help the naïve students understand the SDL process well [18]. Self-directed learning desire, competence, responsibility, and self-regulation required for self-directed learning are not the same for all students, so teacher-assisted learning is necessary to facilitate the process [19]. SDL is considered to be more than a teaching strategy; it is a set of skills that can be taught, learned, and acquired, so as to imbibe the philosophy of lifelong learning [20].

Limitation of the study

The sample size was small. A cross-over study with a larger sample size would have given more conclusive results.

## Conclusions

The present study observed that the traditional teaching method by demonstration/prosection in learning the gross anatomy of an important viscus, viz., the liver, was very satisfactory, yielding a mean post-test score of 8.92 out of 10 marks. Also, the mean post-test score after a session of structured SDL for the liver under the supervision of teachers was 8.53 out of 10. Therefore, it can be said that structured SDL under the supervision of teachers was almost as good as the traditional teaching method by demonstration/prosection in learning the gross anatomy of a viscus. In the context of the reduced number of teachers of anatomy and an increasing number of students, this may open up an option of teaching some portion of gross anatomy, like a few viscera, by SDL. They can be motivated and trained to be lifelong learners. However, a more robust study with a larger sample size can be more conclusive.

## Appendices

Pre-test MCQs (1 × 10 = 10 marks)

1. The liver is located in the _________ and extends _______________ into the ___________.

(a) Left upper quadrant, across the midline, right upper quadrant

(b) Left lower quadrant, across the midline, right lower quadrant

(c) Right upper quadrant, across the midline, left upper quadrant

(d) Right upper quadrant, across the midline, right lower quadrant

2. The liver is an ___________________ organ.

(a) Extraperitoneal

(b) Intraperitoneal

(c) Interperitoneal

(d) Retroperitoneal

3. The area of the liver not covered by the peritoneum is called the _________ __________.

4. The liver is completely covered by a dense fibroelastic connective tissue layer called ______________ __________.

5. The bare area of the liver is located on the _______________ surface of the liver.

(a) Inferior

(b) Anterior

(c) Posterior

(d) Superior

6. The right lobe of the liver is separated from the left lobe by the _______________ _____________ on its diaphragmatic surface.

(a) Main lobar fissure

(b) Falciform ligament

(c) Ligamentum venosum

(d) Coronary ligament

7. The hepatoduodenal ligament contains which structures?

(a) Portal vein

(b) Hepatic artery

(c) Common bile duct

(d) All of the above

8. The round ligament of the liver is also known as:

(a) Ligamentum teres

(b) Ligamentum venosum

(c) Falciform ligament

(d) Triangular ligament

9. Which structure does not enter through porta hepatis?

(a) Portal vein

(b) Common bile duct

(c) Hepatic artery

(d) Hepatic vein

10. The caudate lobe is separated from the right lobe by the …

(a) Ligamentum venosum

(b) IVC

(c) Aorta

(d) Gallbladder fossa

Post-test MCQs (1 ×10 = 10 marks)

1. The liver is located in the _________ and extends _______________ into the ___________.

(e) Left upper quadrant, across the midline, right upper quadrant

(f) Left lower quadrant, across the midline, right lower quadrant

(g) Right upper quadrant, across the midline, left upper quadrant

(h) Right upper quadrant, across the midline, right lower quadrant

2. The liver is an ___________________ organ.

(e) Extraperitoneal

(f) Intraperitoneal

(g) Interperitoneal

(h) Retroperitoneal

3. The area of the liver not covered by peritoneum is called the _________ __________.

4. The liver is completely covered by a dense fibroelastic connective tissue layer called ______________ __________.

5. The bare area of the liver is located on the _______________ surface of the liver.

(e) Inferior

(f) Anterior

(g) Posterior

(h) Superior

6. The right lobe of the liver is separated from the left lobe by the _______________ _____________ on its diaphragmatic surface.

(e) Main lobar fissure

(f) Falciform ligament

(g) Ligamentum venosum

(h) Coronary ligament

7. The hepatoduodenal ligament contains which structures?

(a) Portal vein

(b) Hepatic artery

(c) Common bile duct

(d) All of the above

8. The round ligament of the liver is also known as:

(e) Ligamentum teres

(f) Ligamentum venosum

(g) Falciform ligament

(h) Triangular ligament

9. Which structure does not enter through porta hepatis?

(i) Portal vein

(j) Common bile duct

(k) Hepatic artery

(l) Hepatic vein

10. The caudate lobe is separated from the right lobe by the …

(m) Ligamentum venosum

(n) IVC

(o) Aorta

(p) Gallbladder fossa
